# Supplementary material for: Enhancing the performance and interpretability of epigenetic clocks
Source: Nucleic Acids Res. 2026 Jul 7;54(13):gkag661. doi: 10.1093/nar/gkag661 (PMC13338713; doi:10.1093/nar/gkag661)
Supplement: gkag661_Supplemental_Files [file gkag661_supplemental_files.zip › 290626080452_Supplementary_files.pdf]

Supplementary text

While DNA methylation is frequently associated with the silencing of transposable elements (TEs) [1, 2], several studies have also indicated that TEs may serve as regulatory hubs harboring a plethora of TFBSs [3]. To investigate the age-related dynamics in this specific context, we overlapped TEs with TFs that are enriched and depleted in age-correlated CpGs, respectively. While LINE and SINE elements were depleted for both age-associated of TFs, long terminal repeats (LTRs) enriched TFBSs with age-resilient CpGs **(Supplementary Fig. 13)**. A possible explanation of this could be that TFBS coincide with LTRs are maintained at higher levels of methylation to keep them transcriptionally silenced by genomic mechanisms, otherwise leads to catastrophic repeat activation [1].

The average model coefficients for the 303 age-predictive CpGs ranged from $+0.72 \leq \beta \leq -0.55$, with a median value of -0.01 **(Supplementary Fig. 10)**. The most negative coefficient (-0.55) was found for the singleton cluster associated with the CpG cg22982767. The locus is associated with the PRR34-AS1 gene. The most positive coefficient was obtained for cg16867657 associated with the ELOVL2. Eighty-nine of these even had a statistically significant correlation (adjusted p-value < 0.05, 33.3%)—suggesting a potential role of selected CpGs in gene transcriptional regulation during aging.

Supplementary References

1. Kaluscha S, Domcke S, Wirbelauer C et al. Evidence that direct inhibition of transcription factor binding is the prevailing mode of gene and repeat repression by DNA methylation. Nat Genet 2022; 54: 1895–906. https://doi.org/10.1038/s41588-022-01241-6

2. Ohtani H, Liu M, Zhou W et al. Switching roles for DNA and histone methylation depend on evolutionary ages of human endogenous retroviruses. Genome Res 2018; 28: 1147–57. https://doi.org/10.1101/gr.234229.118

3. Hermant C, Torres-Padilla M-E. TFs for TEs: the transcription factor repertoire of mammalian transposable elements. Genes Dev 2021; 35: 22–39. https://doi.org/10.1101/gad.344473.120

Supplementary figures

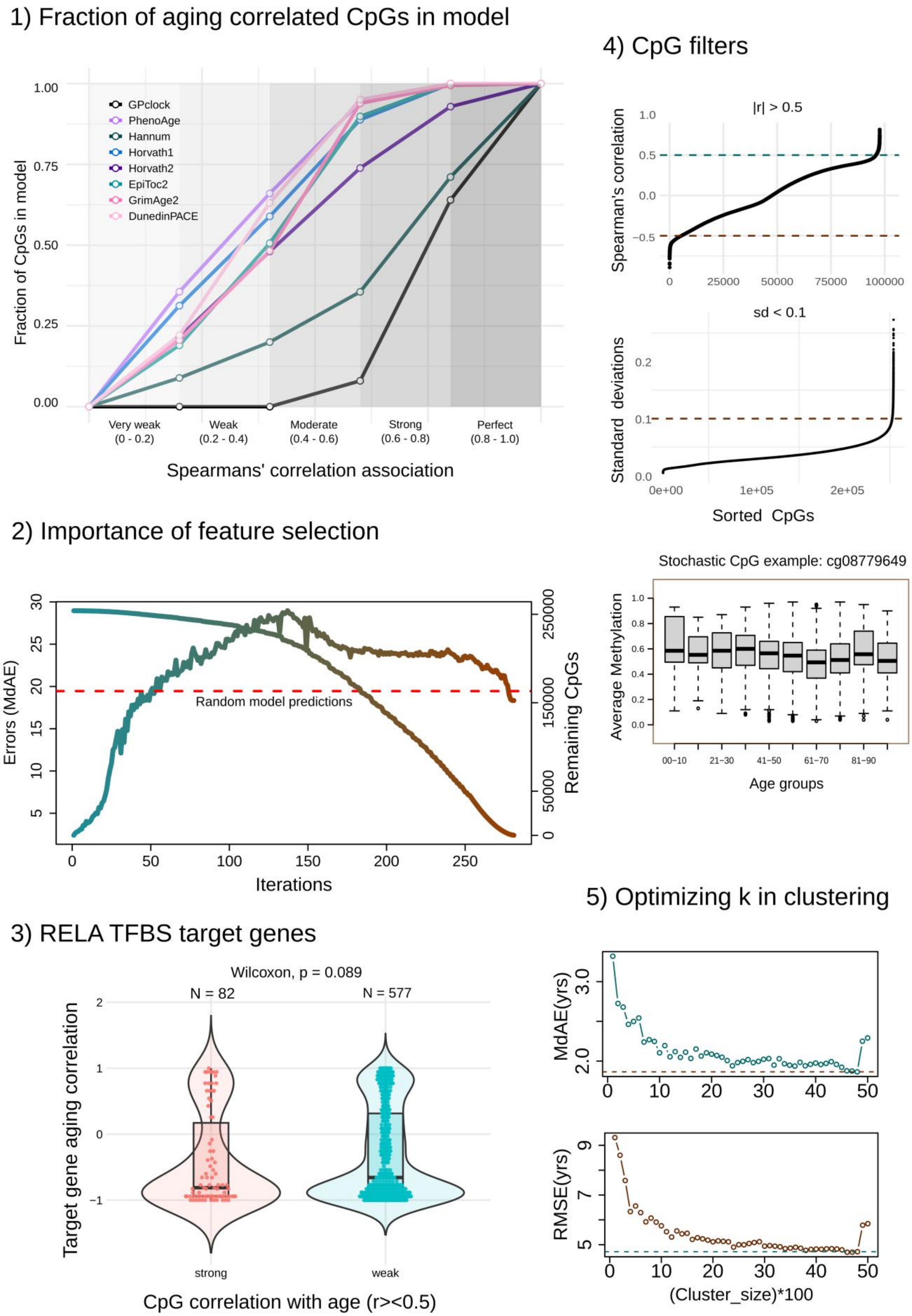
1) Fraction of aging correlated CpGs in model
GPclock
PhenoAge
Hannum
Horvath1
Horvath2
EpiToc2
GrimAge2
DunedinPACE
Fraction of CpGs in model
1.00
0.75
0.50
0.25
0.00
Very weak (0 - 0.2)
Weak (0.2 - 0.4)
Moderate (0.4 - 0.6)
Strong (0.6 - 0.8)
Perfect (0.8 - 1.0)
Spearmans' correlation association
2) Importance of feature selection
Errors (MdAE)
Remaining CpGs
Random model predictions
Iterations
3) RELA TFBS target genes
Wilcoxon, p = 0.089
N = 82
N = 577
Target gene aging correlation
strong
weak
CpG correlation with age (r><0.5)
4) CpG filters
|r| > 0.5
Spearman's correlation
sd < 0.1
Standard deviations
Sorted CpGs
Stochastic CpG example: cg08779649
Average Methylation
Age groups
5) Optimizing k in clustering
MdAE(yrs)
RMSE(yrs)
(Cluster_size)*100

6) Cohort distribution

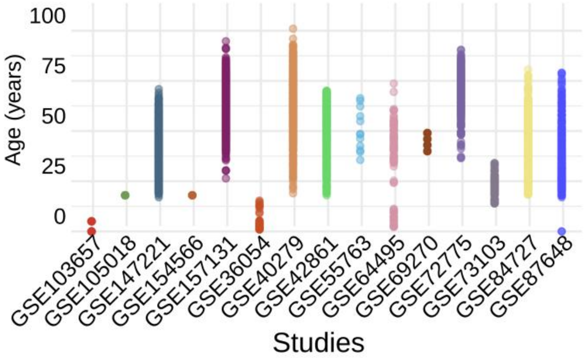

7) CpGs per cluster

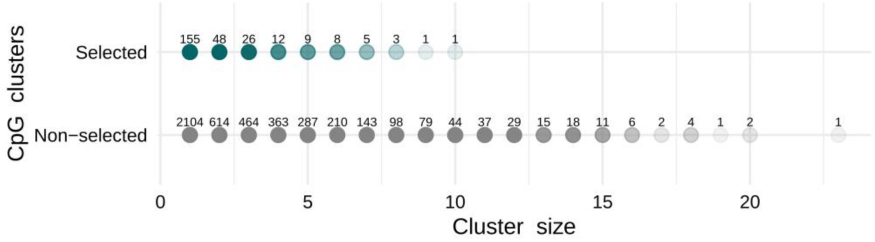

8) CpG overlap clocks

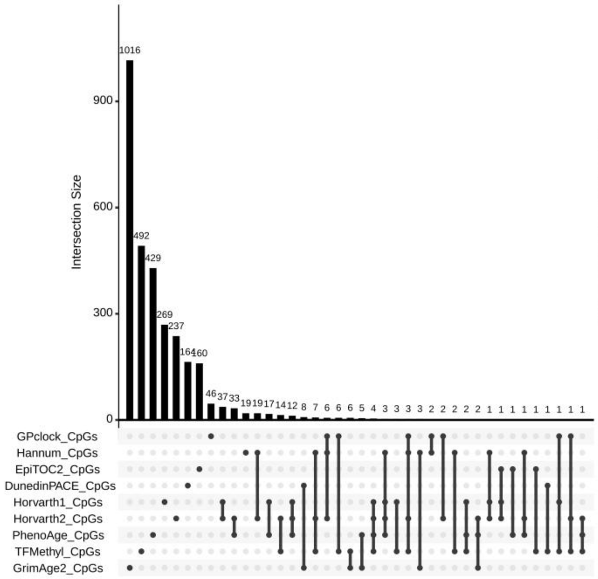

9) CV CpGs TFBS enrichment

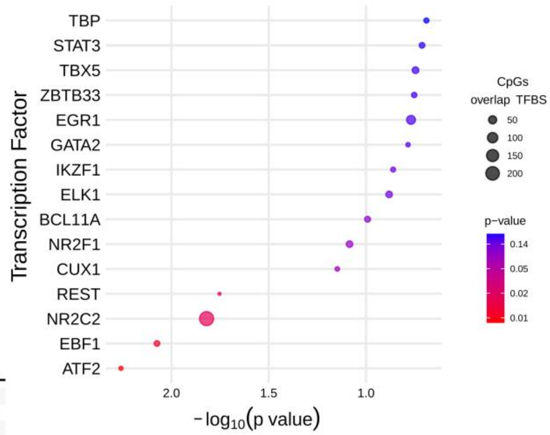

10) CpG::Target-gene methylation::expression

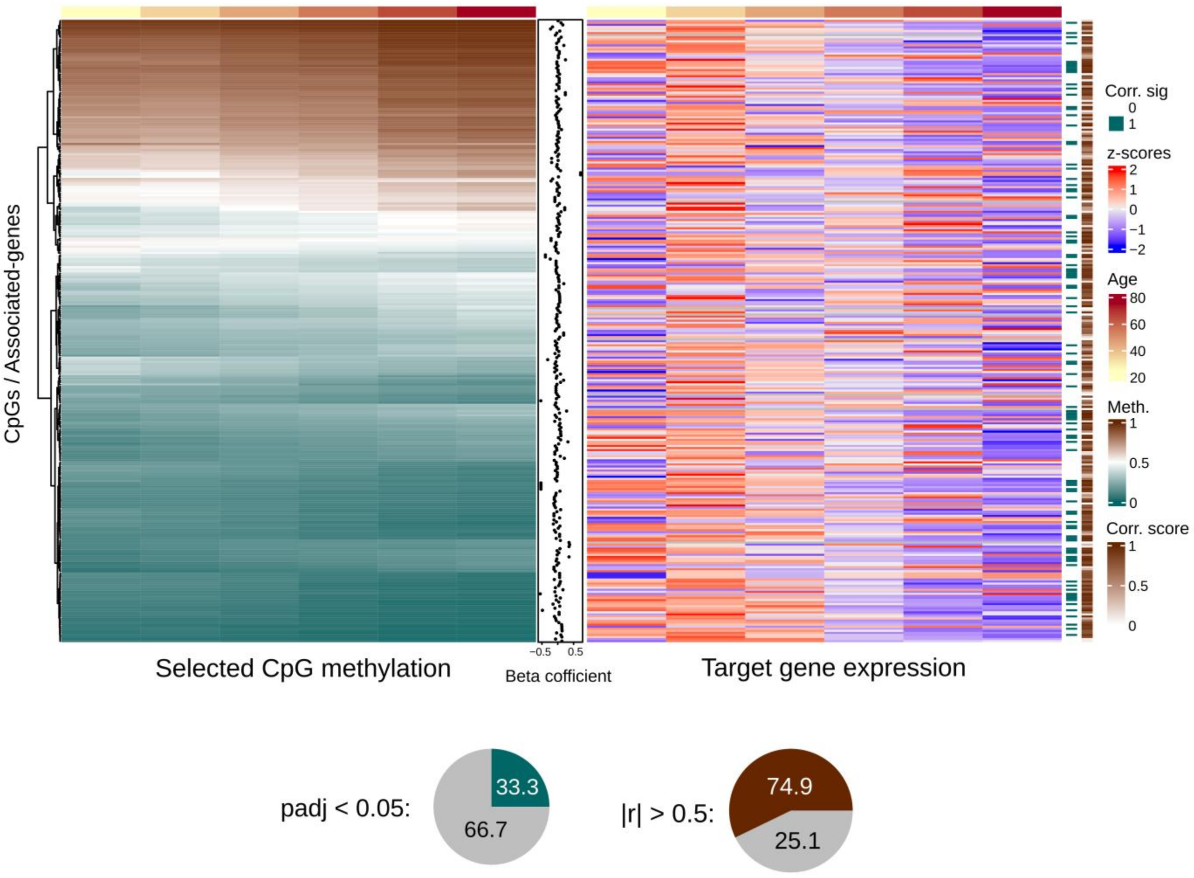

11) DNAm trajectories for gene clusters

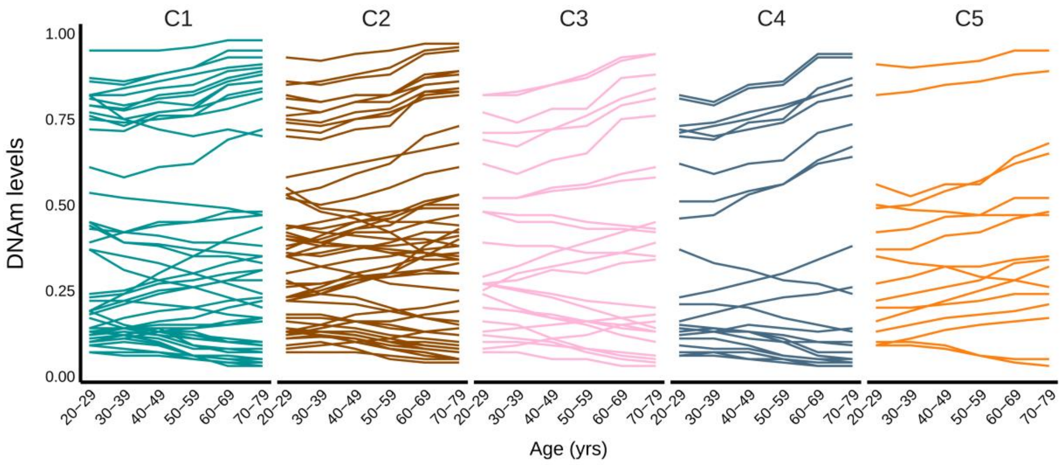

12) Selected loci DNAm and gene expression plots

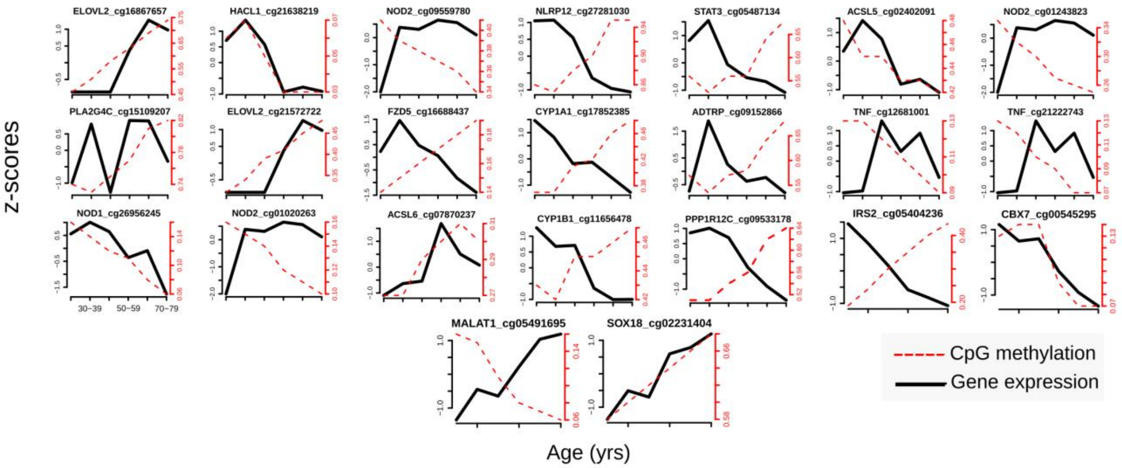

13) Enriched and depleted TFs for TE enrichment

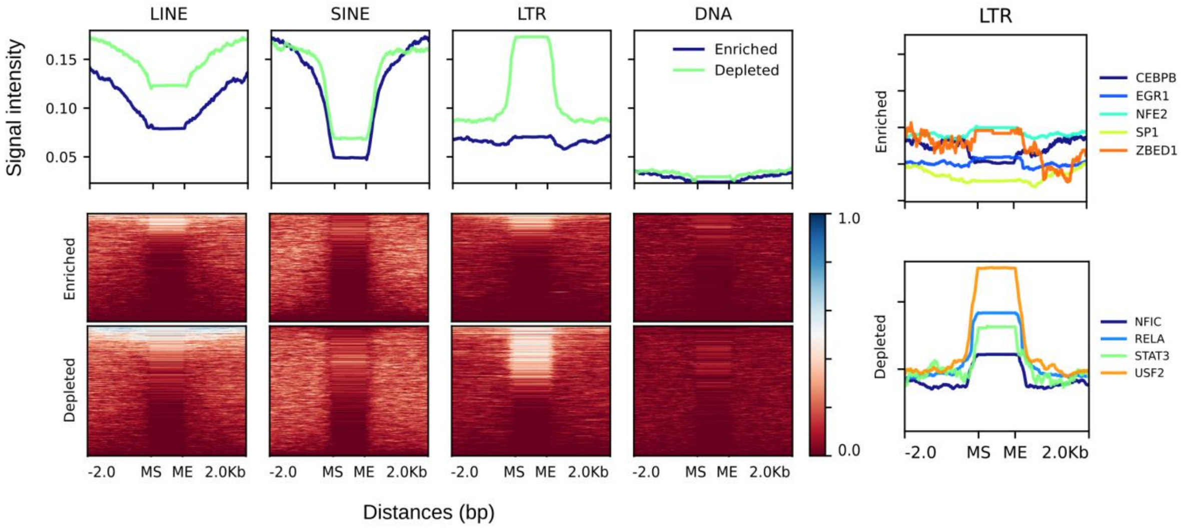

14) Mortality analysis

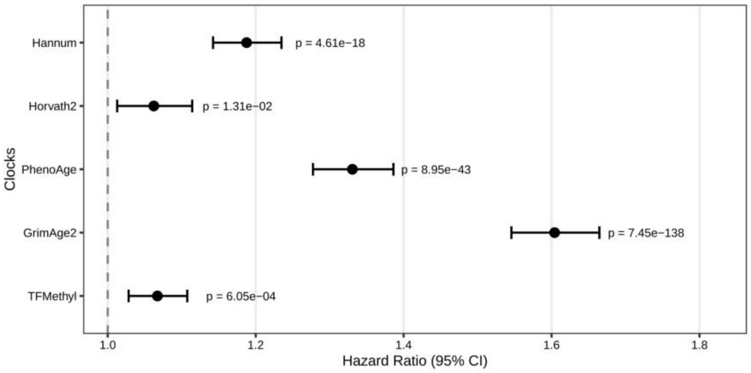

Supplementary Figure. 1) CpG overlaps across aging clocks at different correlation thresholds. Proportion of CpGs from each epigenetic clock retained below increasing correlation cutoffs. Shaded regions denote different correlation intervals. Lines represent eight clocks: EpiTOC2, GP-clock, Hannum, Horvath1, Horvath2, PhenoAge, GrimAge2, and DunedinPACE **2)** Iterative elimination of age-predictive CpGs while training epigenetic age predictors. The plot shows the median absolute error (MdAE) across iterations of elastic net regression models trained on progressively reduced CpG sets. In each round, CpGs with non-zero model coefficients were removed, refining the feature space. The dashed red line indicates the baseline error when predicting all samples as the cohort average, i.e random model. The overlay (right axis) shows the cumulative number of CpGs eliminated over iterations, highlighting the model's reliance on a shrinking subset of predictive features. **3)** Box-violin plot for gene expression correlations of RELA TFBS target genes categorized based on associated CpG methylation change with age. Left is stronger change with age ($|r| > 0.5$) and right is weaker correlations with age ($|r| < 0.5$). **4)** First (from top): The correlation filter ($|r| > 0.5$) for CpGs demonstration. Every CpG with spearman's correlation value above red and blue dotted was further selected. Second: The stochasticity filter for CpGs. Everything above 0.1 red dotted line was removed from the further analysis. Third: Boxplots for an example CpG which was removed. Boxes represent the mean and 95% confidence interval for methylation level at the given loci for a particular age group. **5)** Performance of age prediction across cluster sizes. Median absolute error (MdAE) and root mean square error (RMSE) are plotted against increasing cluster sizes used in optimization. Dashed lines indicate performance at the selected optimal cluster size. **6)** Cohort age distribution for different GSE studies considered in this study. The vertical axis represents the age for the particular sample from each study on the horizontal axis. **7)** Distribution of CpG cluster sizes selected versus not selected by the elastic net model. Each point represents a CpG cluster, colored by whether it was selected by the elastic net model (non-zero coefficients) or not. **8)** Overlap of CpGs across multiple epigenetic clocks. UpSet plot showing shared and unique CpGs between various aging clocks, including EpiTOC2, GP-clock, Hannum, Horvath1, Horvath2, PhenoAge, GrimAge2, and DunedinPACE, and the newly derived TFMethyl Clock model. Bars represent the number of CpGs present in each intersection set. **9)** Top 15 transcription factors enriched among CpG sites consistently selected across 10-fold CV in the aging methylation model. Enrichment was assessed using Fisher's exact test, with point size indicating the number of overlapping CpGs and color representing the p-value on a log scale. **10)** Left: DNA methylation levels at selected CpG sites. Heatmap representing methylation beta values of CpG sites commonly selected across all 10 folds of CV. CpGs were ordered by average model coefficient (weights), shown as a row annotation. Columns represent individual samples, and color intensity indicates methylation level (teal to brown). A top annotation bar shows the chronological age of individuals. Right: Expression of genes associated with selected CpG sites. Heatmap of median gene expression (TPM) per age bin from GTEx whole blood RNA-seq data, converted to z-score normalized across samples. Genes were annotated to CpG sites via the Illumina EPIC v2 annotation and ordered to match the CpG heatmap. Right-side annotations include: Corr. sig as the adjusted significance of Spearman correlation with age (FDR < 0.05, teal); Corr. val as absolute Spearman correlation coefficient $|\rho|$, scaled from white to brown. Bottom: Pie chart showing the percentage of genes with FDR-adjusted p-value < 0.05 from Spearman correlation tests with age (teal) vs. non-significant genes (grey). Pie chart indicating genes with absolute Spearman correlation coefficient greater than 0.5 (brown) versus all others (grey). **11)** Line plots show methylation level dynamics of CpG sites associated with gene clusters C1—C5 across five age bins: 20—29, 30—39, 40—49, 50—59, 60—69, and 70—79 years. **12)** The line plots representing selected loci gene-expression and DNAm change within age-groups from young to old, left to right on the x-axis. The y1 left axis denotes z-scores for gene expression, the y2 axis denotes DNAm level from increasing methylation (bottom to top). **13)** Transposable element signal enrichment around affected and protected TFs. Heatmaps and average signal profiles display the distribution of four major transposable element (TE) classes—LINE, SINE, LTR, and DNA transposons—around TFs either enriched or depleted for methylation-associated signals (±2 kb from motif start or end). Signals were

extracted from corresponding bigWig files using computeMatrix scale-regions, with missing values imputed as zero. The color scale reflects normalized signal intensities (red = high, blue = low), and the average profile above the heatmap shows the mean signal across all regions for each TF class. MS: motif start; ME: motif end. **14)** Forest plot showing hazard ratios (HRs) and 95% confidence intervals for all-cause mortality per unit increase in DNAm-based measures, including Hannum (95% CI: 1.13 - 1.24, p=4.6e-18), Horvath2 (95% CI: 1.00 - 1.10, p=0.0130), PhenoAge (95% CI: 1.28 - 1.39, p=8.9e-43), GrimAge2 (95% CI: 1.53 - 1.67, p=7.45e-138), and TFMethyl (95% CI: 1.03 - 1.11, p=0.0006). Models were adjusted for chronological age and sex. Corresponding p-values are indicated for each clock.

Supplementary tables

Supplementary_table_1.csv: Age-predictive CpGs selected by the Cross-validation models.

The columns in the table represent the following: **1)** Methyl450_Loci = The CpG ID from official illumina 450k array annotation; **2)** Target_gene = The associated target gene for corresponding CpG ID from illumina annotation; **3)** Gene_age_correlation = Correlation of target gene expression (TPM) with chronological age; **4)** p_value = The p-value of the respective gene correlation from spearmans' test; **5)** p_adjusted = Corresponding Benjamini-Hochberg corrected p-value for multiple testing correction; **6)** CpG_weight = Cross-validation averaged beta value for the corresponding CpG; **7)** CpG_chr **8)** CpG_pos **9)** CpG_strand = Chromosomal location of the corresponding CpG.
